# Pupil Detection Algorithm Based on ViM

**DOI:** 10.3390/s25133978

**Published:** 2025-06-26

**Authors:** Yu Zhang, Changyuan Wang, Pengbo Wang, Pengxiang Xue

**Affiliations:** School of Optoelectronic Engineering, Xi’an Technological University, Xi’an 710000, China; zydns6@163.com (Y.Z.); cyw901@163.com (C.W.); xuepx@xatu.edu.cn (P.X.)

**Keywords:** pupil detection, deep learning, MSA, ViM, FFT

## Abstract

Pupil detection is a key technology in fields such as human–computer interaction, fatigue driving detection, and medical diagnosis. Existing pupil detection algorithms still face challenges in maintaining robustness under variable lighting conditions and occlusion scenarios. In this paper, we propose a novel pupil detection algorithm, ViMSA, based on the ViM model. This algorithm introduces weighted feature fusion, aiming to enable the model to adaptively learn the contribution of different feature patches to the pupil detection results; combines ViM with the MSA (multi-head self-attention) mechanism), aiming to integrate global features and improve the accuracy and robustness of pupil detection; and uses FFT (Fast Fourier Transform) to convert the time-domain vector outer product in MSA into a frequency–domain dot product, in order to reduce the computational complexity of the model and improve the detection efficiency of the model. ViMSA was trained and tested on nearly 135,000 pupil images from 30 different datasets, demonstrating exceptional generalization capability. The experimental results demonstrate that the proposed ViMSA achieves 99.6% detection accuracy at five pixels with an RMSE of 1.67 pixels and a processing speed exceeding 100 FPS, meeting real-time monitoring requirements for various applications including operation under variable and uneven lighting conditions, assistive technology (enabling communication with neuro-motor disorder patients through pupil recognition), computer gaming, and automotive industry applications (enhancing traffic safety by monitoring drivers’ cognitive states).

## 1. Introduction

The human eye can reflect a wealth of individual information, including gaze direction and fixation points, fatigue levels, drowsiness states, health conditions, and attention concentration levels [1]. Based on this characteristic, eye movement data have been widely applied in various assistive technologies and physical–mental health monitoring systems [2]. Precisely because of this, pupil localization technology has become a key interdisciplinary research subject.

Pupil localization technology holds extensive application value across multiple critical domains: VFOA (Visual Focus of Attention) tracking [3], driver fatigue monitoring [4], psychological and neuroscientific research [2], consumer behavior analysis [5], virtual reality systems [6], iris recognition technology [7], assistive devices for individuals with disabilities [8], and learning engagement assessment [1]. In medical diagnostics, VFOA tracking technology provides crucial methodology for the early identification of ASD (autism spectrum disorder). As a neurodevelopmental condition typically manifesting during infancy, ASD is characterized by core symptoms including impaired social interaction and persistent attention deficits. Through eye movement trajectory analysis and VFOA monitoring, characteristic behavioral patterns of ASD can be effectively identified. Furthermore, this technology demonstrates significant diagnostic value for neurological disorders, such as aiding Parkinson’s disease diagnosis [9].

The advancement of imaging technology and computational methods has significantly improved pupil detection technology. Traditional manual measurement and basic image processing algorithms are gradually being replaced by methods utilizing machine learning and computer vision. These new technologies provide higher accuracy and robustness, making real-time pupil tracking possible in various environments.

Since 2024, neural networks with Mamba architecture [10] have been widely used in the field of NLP. Compared to Transformer architecture [11], Mamba has higher training and inference efficiency. The Mamba model is based on the SSM (State Space Equation) [12] and can not only be transformed into Transformer form, but also run in a structure similar to RNNs [13], appearing as a linear Transformer rather than traditional QK matrix multiplication. This linear complexity brings attraction to Mamba and allows for parallel training and high throughput.

In recent years, a large number of studies have introduced the Mamba model into the field of computer vision and achieved remarkable results, such as the ViM model [14]. Therefore, we wanted to know if applying Mamba to pupil localization will produce better results.

Through an analysis of outstanding pupil detection models in recent years, it can be observed that the primary challenge in pupil detection tasks remains the high sensitivity to varying lighting conditions and partial occlusions (such as glasses, eyelashes, etc.) in input images, leading to unstable detection accuracy across different datasets. Existing models typically address this issue from the following three perspectives: The first approach involves incorporating channel or spatial attention mechanisms [15,16], enabling the model to adaptively focus on the pupil region. However, the additional attention mechanisms increase the computational complexity of the network itself. The second solution employs Transformer-based architectures or deep convolutional neural networks (e.g., YOLOv8) as backbone networks, leveraging the MSA mechanism’s ability to capture global image information and the expanded receptive fields in intermediate layers of deep convolutional networks [17,18,19,20] to precisely locate the pupil center. Nonetheless, this method significantly raises the computational complexity of the model. The third strategy designs the detection process as a two-stage pipeline [21,22]: the first stage coarsely localizes candidate pupil regions, and the second stage refines the regression of these candidate areas based on the initial coarse results to obtain accurate pupil center coordinates. The drawback of this approach is that if the initial coarse localization deviates slightly, the final regression results will be suboptimal.

To address the aforementioned issues and solutions, this paper adopts a learnable weighted feature fusion method, introducing learnable weights corresponding to the number of patches. Without significantly increasing model complexity, this approach enables the network to adaptively focus on potential pupil regions, maintaining high-precision pupil detection under varying lighting conditions and partial occlusions (essentially, the introduction of learnable weights achieves a similar effect as attention mechanisms, since both channel and spatial attention modules output weights assigned to intermediate features—however, the computational cost of attention mechanisms far exceeds that of simply concatenating learnable weights to patch inputs). Furthermore, this paper is the first to introduce the ViM model into the field of pupil detection. First, leveraging ViM’s long-sequence modeling capability, it effectively overcomes the performance bottlenecks of existing methods under varying lighting and partial occlusion conditions. Second, compared to ViT [23], the SSM-based ViM architecture significantly reduces computational complexity, enabling real-time detection while maintaining high accuracy. Finally, if computational efficiency and resource consumption are disregarded, the pairwise interaction property of ViT’s self-attention mechanism grants it superior global modeling capability compared to ViM (precisely because this pairwise matrix multiplication in MSA leads to ViT’s high computational complexity). To address this, this paper combines ViM with ViT’s self-attention mechanism, which excels at fine-grained global context modeling, and integrates MSA with ViM while employing FFT to reduce the computational overhead of MSA’s matrix operations. This fusion ultimately forms the proposed ViMSA model, offering a novel solution for pupil detection and advancing the application and development of the Mamba architecture in computer vision.

The main contributions of this paper are as follows:

- Enhanced and extended ViM, proposing the ViMSA model.
- Weighted Feature Fusion, a learnable weighting mechanism, is introduced to enhance the model’s adaptive learning capability for effective information extraction, thereby reducing its sensitivity to varying illumination conditions and partial occlusions.
- Integration of FFT-accelerated MSA enables ViM to maintain low computational complexity while acquiring ViT’s more refined global modeling capability.
- Extensive experiments on multiple pupil datasets demonstrate the superior performance of our proposed method.

The remainder of this paper is organized as follows: Section 2 presents the background and related work on pupil center localization and the ViM model. Section 3 elaborates on the construction of the proposed ViMSA model. Section 4 provides detailed experimental results of the proposed method. Section 5 discusses this work, highlighting the advantages and applications of the ViMSA model while discussing potential limitations. Finally, Section 6 concludes this research.

## 2. Related Works

### 2.1. Pupil Detection

We reviewed major research advances in pupil detection methodologies. Existing pupil localization methods can be primarily categorized into learning-based and non-learning-based approaches [24].

Non-learning-based pupil detection methods refer to traditional algorithms applied in early-stage research, which primarily include edge detection, threshold segmentation, feature extraction, and template matching. Edge detection-based algorithms identify potential pupil boundaries by analyzing brightness gradients in pupil images and then filter the final boundaries based on predefined conditions. The Wolfgang Fuhl team has proposed two edge filtering algorithms based on ellipse estimation, ExCuSe [25] and ElSe [26], both of which combine edge detection, ellipse fitting, and geometric constraints to locate the pupil. However, they rely on manually designed features and have poor robustness. Thiago Santini et al. [27] proposed PuReST based on ExCuSe and ElSe, combined with Kalman filtering to improve the stability of continuous frames. However, it is susceptible to different lighting conditions and has poor robustness. Threshold segmentation-based methods binarize images using thresholds to enable pupil detection. Professor Changyuan Wang’s team [28] first binarized pupil images and then applied Hough transform to detect circles and ellipses for pupil center localization, while this method features relatively low complexity, its accuracy heavily depends on threshold selection, with improper thresholds leading to performance degradation. Feature extraction-based approaches detect pupils by analyzing specific characteristics like size, gradient, and grayscale. Timm F. et al. [29] determined pupil centers by computing gradient vector fields, demonstrating certain robustness to illumination variations, though computational complexity increases significantly with higher image resolutions. Template matching-based techniques utilize predefined pupil models to match pupil regions in images. Cerrolaza et al. [30] constructed pupil models using point distribution methods and searched for actual pupil positions through template matching, this method exhibits high computational complexity and requires substantial prior knowledge of pupil characteristics. In summary, the challenges that traditional algorithms generally need to address are still high sensitivity to lighting and environment, complex computation, and the need for a large amount of prior knowledge of pupil models, resulting in a high false detection rate.

Unlike non-learning-based methods, learning-based approaches leverage popular machine learning models in recent years to extract effective discriminative features from pupil images for precise pupil localization. These models excel at extracting deep semantic features and their interrelationships from images. Krafka et al. [31] proposed the iTracker model for mobile pupil detection, which employs a lightweight CNN architecture to extract pupil features and perform recognition. However, this model inherits CNN’s tendency to over-focus on local features, limiting its detection accuracy. Larumbe-Bergera et al. [32] introduced a ResNet50-based pupil detection network that incorporates global pooling layers to compensate for CNN’s neglect of global features, but the excessive use of fully connected layers increases the network’s computational complexity. Yiu et al. [33] proposed a fully convolutional network model capable of segmenting the entire pupil region and locating the pupil. However, their model is based on the prior assumption that the pupil is circular, which imposes certain limitations. Vera-Olmos et al. [34] utilized deep convolutional networks for pupil tracking, integrating dilated convolutions to expand the receptive fields between convolutional layers and adopting a feature pyramid structure to combine shallow and deep image features for pupil localization. Nevertheless, this method suffers from substantial model parameters and high complexity. Wolfgang Fuhl et al. [35] proposed a two-stage detection model, PupilNet v2.0, where the first stage performs coarse localization and the second stage refines the regression. However, the final detection accuracy is affected by the initial coarse localization results.

Gorkem Can Ates et al. [15] employed ResNet integrated with SE (Squeeze-and-Excitation) attention modules, achieving superior detection performance on low-resolution images while experiencing decreased efficiency and accuracy in high-resolution scenarios. Kejuan Xue et al. [16] based their work on YOLOv8n (the least complex model in the YOLOv8 series), incorporating a dual attention mechanism to enhance the model’s ability to capture pupil region information under varying lighting conditions and introducing acyclic convolutions in the encoding part to reduce model complexity. However, the matrix multiplication operations in the spatial and channel attention mechanisms still increase the overall computational load. Chugh, S. et al. [17] enhanced the UNet architecture by incorporating CSA (Contrastive Self-Attention Mechanism) to capture global features in pupil images, though the matrix operations involved in this mechanism, similar to standard self-attention, increase computational complexity. H.M. Zhang et al. [18] integrated FPN (Feature Pyramid Network) with ViT to mitigate invalid signal interference caused by occlusions, but the introduction of ViT significantly elevates the model’s computational demands. Zhuohao Guo et al. [19] selected YOLOv8 as the network backbone and introduced deformable convolutions to adapt the model’s learning direction to the pupil region, improving detection accuracy but increasing model complexity. Wang Li et al. [20] introduced a hybrid architecture combining CNN-based ResNeSt modules [36] with Transformers, merging CNN’s local feature extraction capability with Transformer’s global feature capture ability to improve pupil detection accuracy, though the model complexity remains high. Jian Xun Mi et al. [21] employed a two-stage voting mechanism with a fully convolutional network to regress the pupil center point offset in a top-down manner for pupil center localization. B. Zhu et al. [22] proposed a hybrid approach integrating deep learning with traditional algorithms, where the first stage employs the YOLOv5s deep neural network for coarse pupil localization, followed by the second stage utilizing the Canny edge detector for pupil contour extraction, ultimately achieving accurate pupil positioning. However, as a two-stage model similar to PupilNet v2.0, its prediction accuracy is susceptible to the initial coarse localization results. Gabriel Bonteanu et al. [37] used two structurally identical fully convolutional networks to predict the pupil’s x and y coordinates separately, resulting in lower computational costs. However, explicitly separating the x and y coordinates of the pupil leads to temporal inconsistency in the results, as there is an implicit relationship between the x and y coordinates at any given moment. Genjian Yang et al. [38] adopted a ResNet-based architecture with dilated convolutions to address potential inconsistencies in pupil size within images, but the model remains vulnerable to environmental lighting conditions.

### 2.2. ViM

Deep learning models have demonstrated remarkable performance across various artificial intelligence tasks. To date, diverse deep learning architectures have emerged, with model structures fundamentally determining their functionalities. Typically, the initially proposed MLP (Multilayer Perceptron) [39], also known as fully connected networks, can learn deep representations of data. Subsequently, developed CNN primarily serve computer vision tasks by extracting local image features. GANs (Generative Adversarial Networks) [40], as generative architectures, find applications in both sequential and image domains. Comprising a generator and discriminator that compete adversarially, they achieve mutual improvement through this minimax game during training. GNNs (Graph Neural Networks) [41] specialize in processing graph-structured data. RNNs (Recurrent Neural Networks) are primarily used for natural language processing tasks due to their sequential data processing capabilities. However, they suffer from vanishing/exploding gradient problems with long sequences, failing to capture relationships between distant tokens [42]. Although subsequent improvements like GRUs (Gated Recurrent Units) [43] and LSTM (Long Short-Term Memory) networks [44] mitigate these gradient issues, they cannot provide fundamental solutions. In 2017, the Transformer architecture was introduced, which utilizes self-attention mechanisms to process data, effectively addressing the vanishing/exploding gradient problems inherent in RNNs when handling long sequences. The Transformer has subsequently been adapted to computer vision tasks, as exemplified by ViT [18]. Compared to CNN, ViT demonstrates superior capability in capturing global image information, albeit at the cost of significantly increased computational overhead. The incorporation of self-attention mechanisms results in quadratic computational complexity growth relative to image resolution in Transformer-based models. SSM also serve as alternative approaches for sequence processing tasks, though they similarly suffer from high computational demands. The S4 model [45] introduced convolutional operations to enable parallel training, but its fixed parameterization imposes limitations on generalization across diverse input data types. Building upon S4, the Mamba model was developed, which employs hardware-aware parallel scan algorithms to substantially accelerate training efficiency while implementing input dependent parameter selection mechanism. Given Mamba’s success in sequential tasks, researchers have extended it to computer vision, yielding ViM models analogous to ViT.

The ViM model divides input images into uniformly sized patches, which are then processed as sequential inputs during training. However, for pupil detection tasks, the contribution of each segmented pupil patch to the final detection result varies significantly. Treating all patches equally limits further improvements in detection accuracy. To address this, we introduce a weighted feature fusion method that assigns learnable weights to patch sequences, enabling ViM to adaptively adjust each patch’s contribution to the detection outcome. Moreover, while mutual information between different regions of pupil images is crucial for accurate detection, the 1D convolutional modules in ViM primarily capture local feature relationships, neglecting long-range dependencies among patches. We replace these 1D convolutions with the MSA module from Transformers to enhance ViM’s ability to model long-range interactions. Additionally, we employ FFT to reduce the computational complexity of MSA, thereby improving the model’s operational efficiency.

## 3. Methodology

### 3.1. ViM Model

**Figure 1:**
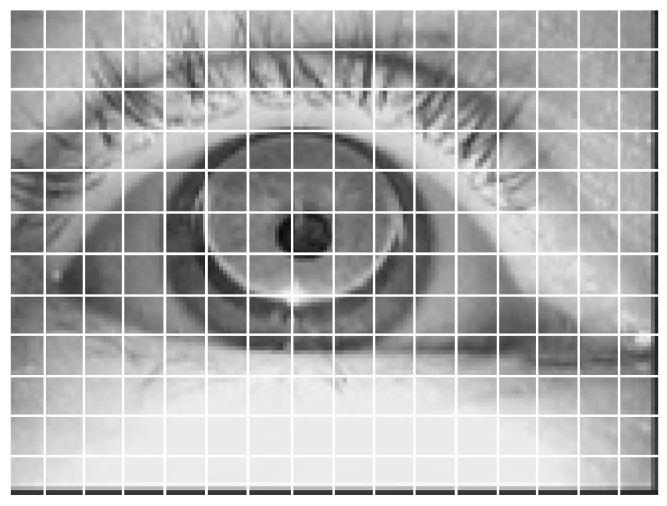
Example of dividing an image into patches.

The standard Mamba model is designed for one-dimensional sequential data. To process 2D image data $i\in {\mathbb{R}}^{h\times w\times c}$(where h and w denote height/width and c represents channel depth), the image is first partitioned into flattened 2D patches $i_{p}\in {\mathbb{R}}^{J\times \left(p^{2}\times c\right)}$ (with J being the patch count and p the patch side length). As shown in Figure 1, each white bounding box in the image corresponds to one patch.

**Figure 2:**
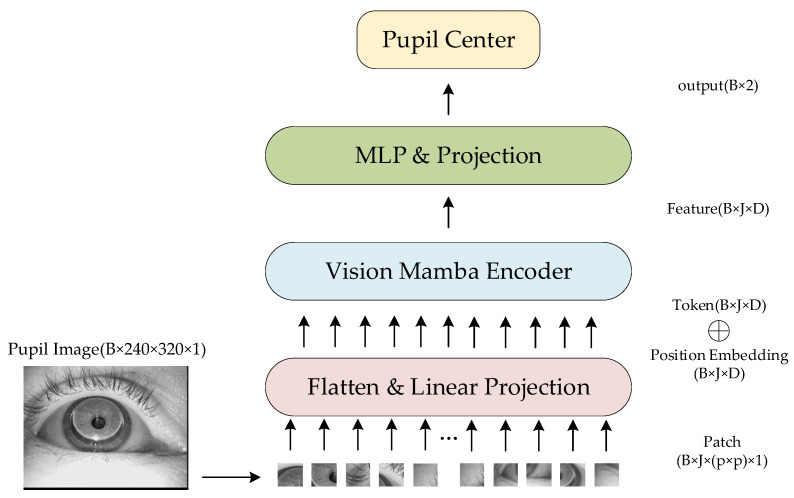
ViM model.

These patches are then linearly projected via a fully-connected layer into D-dimensional vectors $i_{pd}\in {\mathbb{R}}^{J\times D}$. Finally, positional embeddings $E_{pos}\in {\mathbb{R}}^{J\times D}$ are added to $i_{pd}$, forming the input tensor for the Vision Mamba module. The ViM architecture is illustrated in Figure 2, where the symbol ⊕ represents the element wise addition of the vector.

**Figure 3:**
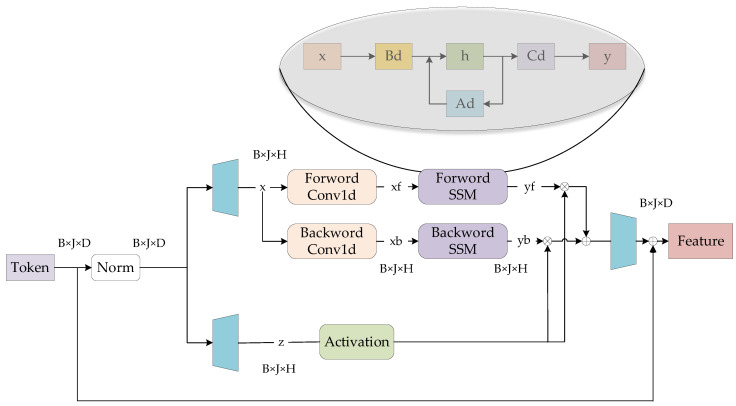
Vision Mamba Encoder structure.

The output dimensionality for each processing step is indicated at corresponding positions on the right side of Figure 1, where B represents the batch size. As the core component of ViM, the Vision Mamba Encoder’s internal architecture is detailed in Figure 3.

In the Vision Mamba Encoder, tokens first undergo normalization (Norm) to prevent gradient vanishing or explosion during training. The normalized tokens then pass through two distinct linear layers (represented by the blue trapezoidal modules on the left in Figure 3) for dimension expansion. Here, H denotes the internal hidden dimension of the linear layer. The expanded x vectors are processed separately through forward and backward pathways, yielding output feature vectors yf and yb, respectively. Simultaneously, the expanded z vectors are activated and then undergo do product with both yf and yb. The results are summed and projected back to the original input dimension through another linear layer (the blue trapezoidal module on the right in Figure 3). The final output is obtained via skip connection. The forward and backward pathways share an identical architecture, with the forward path processing the x sequence in its original order and the backward path operating on the reversed x sequence. The activation module employs the SiLU (Sigmoid Linear Unit) activation function [46], defined by the following equation:

(1) $$\left\{\begin{array}{l} f\left(x\right)=x*\sigma \left(x\right)\\ \sigma \left(x\right)=1/\left(1+e^{-x}\right) \end{array}\right.$$

Additionally, Figure 3 displays the State Space Model (SSM) module adopted in the ViM architecture. The computational process of this module is formally expressed by the following equation:

(2) $$\begin{array}{l} h^{\prime }\left(t\right)=A_{d}h\left(t\right)+B_{d}x\left(t\right)\\ y\left(t\right)=C_{d}h\left(t\right) \end{array}$$

In the equation, $h\left(t\right)$ represents the hidden state of the SSM system at time step, while $h^{\prime }\left(t\right)$ denotes the hidden state at the next time step. The term $x\left(t\right)$ corresponds to the system input, and $y\left(t\right)$ to the system output. The matrix $A_{d}$ (discrete state transition matrix) characterizes the inter-state transition relationships, $B_{d}$ (discrete input matrix) governs how input signals affect the state, and $C_{d}$ (discrete output matrix) defines the mapping from state vectors to output vectors. For practical computation, $A_{d}$, $B_{d}$, and $C_{d}$ are dynamically determined by the input vectors, serving as trainable parameters in ViM. The SSM module employs matrix operations to significantly accelerate both training and inference efficiency.

### 3.2. Weighted Feature Fusion

Pupil detection is different from other computer vision tasks because the distribution of effective information in pupil images is uneven, especially under variable lighting and partial occlusion conditions, and the contribution values of each part of the image to pupil detection are different (from the pupil image, it can be roughly judged that the effective information decreases layer by layer in an elliptical shape from the center of the pupil outward [21]). If the patch sequence obtained after uniform segmentation is treated equally, it will limit the improvement of the model’s detection accuracy under different lighting and occlusion conditions.

**Figure 4:**
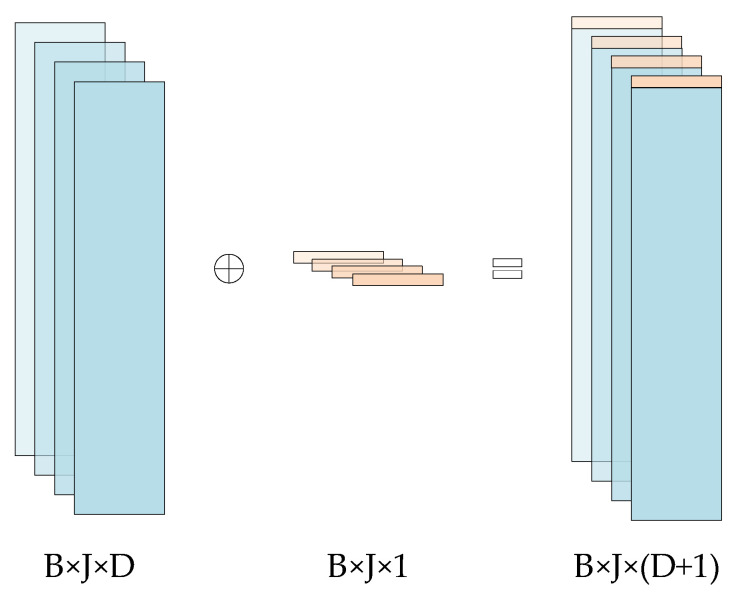
Diagram of Weight Concatenation.

To solve the above problems, this paper adopts a weighted feature fusion method. After cutting the pupil image into patch sequences, a learnable patch weight is concatenated on this basis. The dimension of the concatenated patch sequence is B×J×(D + 1). This weight can weight each patch when mapping features to pupil coordinates, making the ViM model more focused on learning features related to pupil regions. Figure 4 is a schematic diagram of weight concatenation.

In Figure 4, the blue square represents the feature patch vector with dimensions of B×J×D, and the yellow square represents the concatenated weight vector.

Considering the issue of computational efficiency, the number of neurons in the fully connected layer should not be too large. In this paper, a global average pooling module [47] is added before the output layer of the model. After the Vision Mamba Encoder layer, weight removal operations are used to extract weight columns separately, and the extracted residual feature vectors are subjected to Global Average Pooling operations. Finally, the pooled feature vectors are weighted and sent to MLP for pupil center calculation.

### 3.3. ViMSA

In pupil images, various regions contribute to pupil detection and localization to some extent, such as the iris, sclera, and other ocular structural areas, as well as external features like Purtscher’s Spot generated under infrared camera illumination. Therefore, the relationships between different regions in pupil images are crucial for the task of pupil detection and localization. These inter-regional relationships are commonly referred to as global feature representations.

Compared to ViT, ViM employs SSM to capture global dependencies through implicit state transitions and input-dependent recursive mechanisms, demonstrating superior computational efficiency and training effectiveness over ViT. However, since ViM does not explicitly compute pairwise interactions between all patches, its global dependency modeling capability remains slightly inferior to ViT. This raises a critical research question: How can we endow ViM with ViT-level global relational modeling capacity while preserving its efficient detection characteristics?

**Figure 5:**
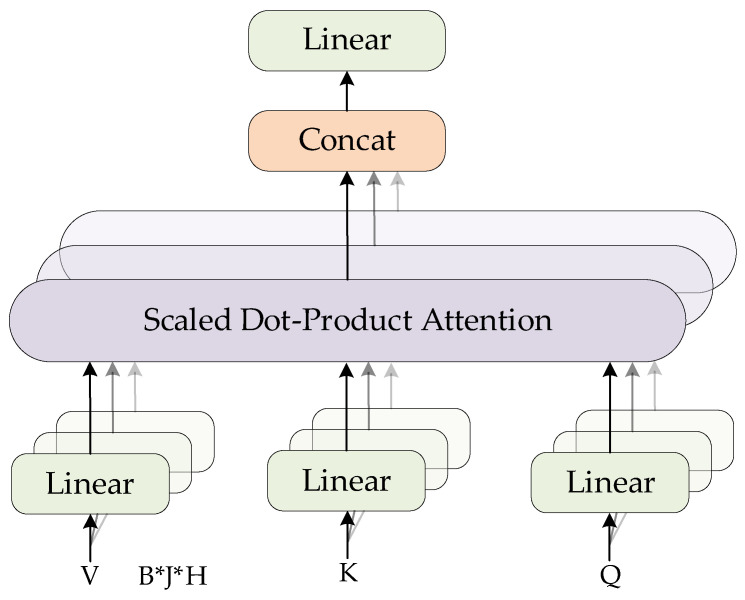
MSA structural diagram.

Through observation, it can be seen that the 1D convolutional module used in the ViM model tends to extract short-range dependencies in sequential data, focusing on local patch relationships, while its ability to capture global dependencies is relatively limited. This becomes a constraint that hinders the ViM model from achieving better performance in pupil detection tasks. In comparison, the MSA mechanism in Transformer can effectively capture global information by modeling relationships across all patches. Its structural diagram is shown in Figure 5.

Here, Q, K, and V are all similar to the feature vector x in ViM, with dimensions of B×J×H. After being projected through linear layers, the Q, K, and V vectors are fed into the Scaled Dot-Product Attention module, whose computation is defined by the following formula

(3) $$Attention\left(Q,K,V\right)=Softmax\left(\frac{Q\cdot K^{T}}{\sqrt{d_{k}}}\right)\cdot V$$

In the formula, $d_{k}$ denotes the number of heads in multi-head self-attention, and · represents matrix multiplication. This matrix operation is fundamentally why the MSA module can effectively capture global relationships. Building on this principle, we replace the 1D convolution in ViM with MSA, thereby enhancing the model’s ability to incorporate global feature representations in its regression output.

While MSA effectively addresses the global dependency problem, it simultaneously introduces a new challenge: high computational complexity. The primary factor contributing to MSA’s computational overhead lies in its high-dimensional matrix multiplication operations.

To address the high computational complexity introduced by MSA, this paper first decomposes the matrix multiplication into a sum of multiple outer products. Given matrix A with dimensions m×n and matrix B with dimensions n×p, the resulting matrix C from their multiplication can be expressed by Equation (4).

(4) $$C=A\times B=\sum_{k=1}^{n}a_{k}⊗b_{k}$$

Here, $a_{k}$ represents the k-th column vector of matrix A, and $b_{k}$ denotes the k-th row vector of matrix B, where ⊗ indicates the vector outer product operation. The outer product is mathematically defined by Equation (5).

(5) $${\left(a⊗b\right)}_{ij}=a_{i}\cdot b_{j}$$

The obtained $a_{k}$ and $b_{k}$ satisfy Equation (6).

(6) $$\psi \left(a_{k}⊗b_{k}\right)=\psi \left(a_{k}\right)*\psi \left(b_{k}\right)$$

Here, ψ denotes the FFT and ∗ represents dot product in the frequency domain. Given a sequence X of length N(denoted as $x\left[n\right]$), its (DFT) Discrete Fourier Transform) is defined by Equation (7).

(7) $$X\left[k\right]=\sum_{n=0}^{N-1}x\left[n\right]e^{-i\frac{2\pi }{N}kn},k=0,1,\cdots ,N-1$$

The FFT improves computational efficiency by leveraging the recursive nature of the DFT. The FFT computation can be decomposed as follows.

(8) $$\left\{\begin{array}{l} X\left[k\right]=X_{even}\left[k\right]+e^{-i\frac{2\pi }{N}k}X_{odd}\left[k\right]\\ X\left[k+\frac{N}{2}\right]=X_{even}\left[k\right]-e^{-i\frac{2\pi }{N}k}X_{odd}\left[k\right] \end{array}\right.$$

Here, $X_{even}\left[k\right]$ represents the DFT of even-indexed elements in the input sequence, while $X_{odd}\left[k\right]$ denotes the DFT of odd-indexed elements.

Qualitatively, for an N×N matrix, standard matrix multiplication requires $N^{3}$ multiplication operations and $N^{3}-N^{2}$ addition operations. By decomposing the matrix multiplication into vector outer products and converting them to frequency–domain dot product via FFT, the computational complexity reduces to only $N^{2}+N\log_{2}N$ multiplications and $N\log_{2}N$ additions. This approach effectively achieves the goal of reducing the module’s computational complexity.

**Figure 6:**
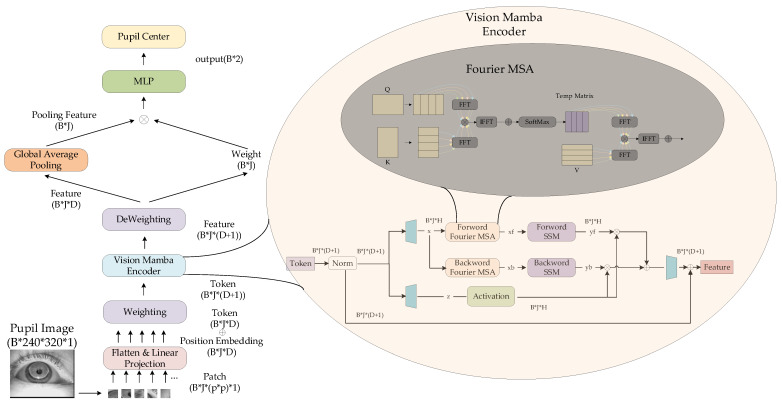
The architecture of ViMSA.

For convenience, we refer to this model as ViMSA. The overall architecture of ViMSA as illustrated in Figure 6.

According to Equation (4), the QK matrix in MSA is decomposed into an outer product form of row and column vectors. Following Equation (6), FFT is applied separately to the decomposed row and column vectors. The resulting vectors are then multiplied, and the obtained matrices are converted back to the time domain through IFFT operations and summed to yield the original matrix multiplication result (denoted as Temp Matrix). The operation between Temp Matrix and V matrix follows a similar process as QK, as illustrated in the Fourier MSA module in Figure 6, where the different colored curves connected to FFT in the Fourier MSA module represent the use of FFT for the row and column vectors of each decomposed matrix.

## 4. Experiments

### 4.1. Data Acquisition

The experiment employed 30 distinct datasets comprising approximately 135,000 pupil images to evaluate the performance of the proposed ViMSA model. Among these, 29 datasets were provided by Wolfgang Fuhl’s research team and were previously cited in ExCuSe [25], ELSe [26], and PupilNet 2.0 [27]. Following the naming convention established in prior researches, these 29 datasets are designated with Roman numerals as DI, DII, DIII, DIV, DV, DVI, DVII, DVIII, DIX, DX, DXI, DXII, DXIII, DXIV, DXV, DXVI, DXVII, DXVIII, DXIX, DXX, DXXI, DXXII, DXXIII, DXXIV, newDI, newDII, newDIII, newDIV, and newDV [48].

The additional dataset used is the publicly available iris dataset CASIA-Iris-Thousand, which contains 20,000 iris images collected from 1000 participants. For convenience in subsequent experiments, this dataset will be referred to as CIT.

**Figure 7:**
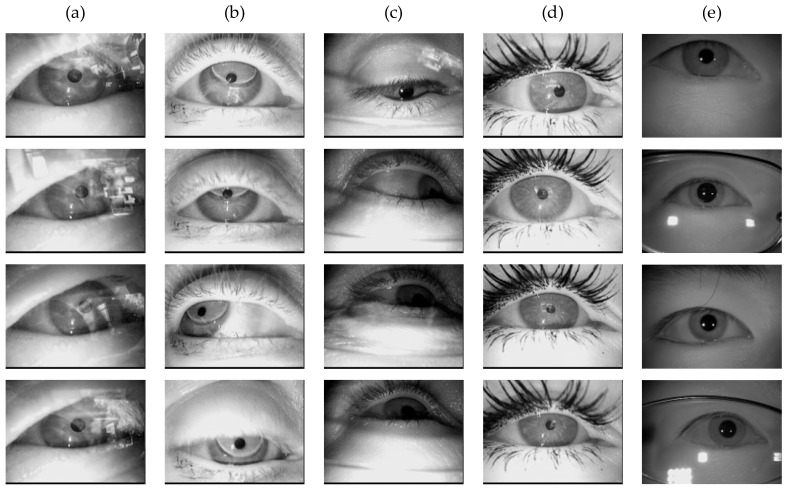
Some image examples from different datasets used for experiments: (**a**) DI—Reflection; (**b**) DV—Contact lenses; (**c**) DII—Bad illumination; (**d**) DVI—Mascara; (**e**) CIT.

Figure 7 presents representative pupil images sampled from these datasets.

In the experiments, all pupil images were single-channel grayscale. The CIT dataset contained images sized 640 × 480, while all other datasets featured 384 × 288 images. For standardization, all images were resized to 320 × 240. Specifically: (1) CIT images were proportionally scaled down, and (2) other datasets underwent center cropping to preserve the 320 × 240 central region. The data were split into 85% for training and 15% for testing.

### 4.2. Experimental Environment

The hardware configuration and runtime parameters used in our experiments are specified in Table 1.

### 4.3. Evaluation Metrics

This paper adopts two classic regression-based evaluation metrics: RMSE (Root Mean Squared Error) and DR (Detection Rate) within 5-pixel error range, which serve as primary performance measures for pupil detection tasks. The RMSE formula is presented below.

(9) $$RMSE=\sqrt{\frac{1}{N}\sum_{i=1}^{N}\left({\left(x_{i}^{\prime }-x_{i}\right)}^{2}+{\left(y_{i}^{\prime }-y_{i}\right)}^{2}\right)}$$

Here, $x_{i}^{\prime }$,$y_{i}^{\prime }$ denote the predicted pupil center coordinates from the model, while $x_{i}$,$y_{i}$ represent the ground truth pupil center coordinates, with N being the total number of pupil images in the test set. A smaller RMSE value indicates higher pupil detection accuracy. The loss function adopted in our model is also RMSE.

The formula for calculating the DR is as follows:

(10) $$DR=\frac{C}{N}\times 100\%$$

Here, N represents the total number of samples, and C denotes the count of correct predictions within the specified pixel tolerance.

### 4.4. Ablation Experiment

**Figure 8:**
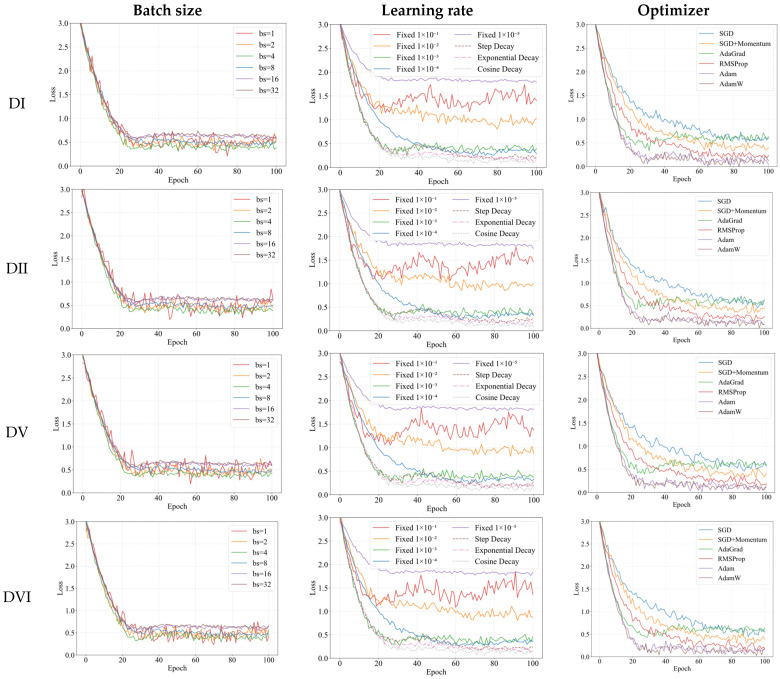

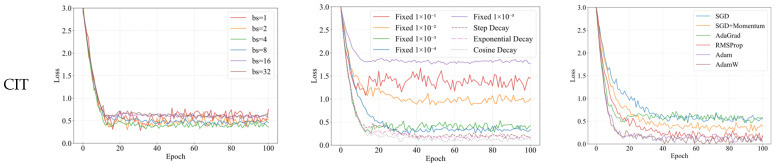
Demonstration of loss curves for ViMSA on partial datasets under different training hyperparameters.

For the experimental configuration regarding batch size, learning rate, and optimizer selection, we first fixed the learning rate at 1 × 10^−3^ and chose Adam as the optimizer. Figure 8 demonstrates the convergence behavior of the model under different batch sizes across various datasets, while Table 2 presents the corresponding DR5 (detection rate within 5-pixel error) in % (with fixed model parameters: 8 MSA heads, 10 × 10 patch size, and Gaussian initialization for the weights in weighted fusion). In Table 2, the first column lists the dataset names, the second column shows the corresponding data volumes, and the last column provides brief descriptions of each dataset.

Figure 8 reveals that when the batch size is relatively small (bs = 1 and bs = 2), although the model can converge to favorable values, the training process exhibits excessive fluctuations due to the gradient noise introduced by smaller batches, which helps the model escape poor local optima. Conversely, with larger batch sizes (bs = 16 and bs = 32), while the training process remains stable, the model fails to converge to better solutions, indicating it becomes trapped in inferior local optima—essentially overfitting to the training data—as the reduced inter-batch information variation diminishes necessary noise, preventing the model from escaping suboptimal solutions. Overall, when bs = 4, the model demonstrates relatively stable training while achieving superior convergence. Notably, across all batch sizes, the model converges significantly faster on the CIT dataset compared to others, likely because CIT, unlike the remaining 29 datasets, does not intentionally incorporate extreme environmental conditions (e.g., strong reflections, contact lenses, or mascara occlusion), making it inherently less challenging for detection. As shown in Table 2, a batch size of 4 yields superior detection accuracy across most datasets. Therefore, bs = 4 is selected for subsequent experiments.

With the batch size fixed at 4 and Adam selected as the optimizer, Figure 8 illustrates the model’s convergence behavior under different learning rate strategies, while Table 3 presents the corresponding DR5. The evaluated learning rate configurations include: (1) fixed rates (1 × 10^−1^, 1 × 10^−2^, 1 × 10^−3^, 1 × 10^−4^, 1 × 10^−5^); (2) Step Decay (1 × 10^−3^ for first 40 epochs, 1 × 10^−4^ for next 40 epochs, 1 × 10^−5^ thereafter); (3) Exponential Decay (γ = 0.95, initial rate = 1 × 10^−3^); and (4) Cosine Decay (cycle = 100, initial rate = 1 × 10^−3^, minimum rate = 1 × 10^−5^).

Figure 8 demonstrates that with a fixed learning rate of 1 × 10^−5^, the model’s loss barely decreases due to insufficient parameter updates from excessively small gradients. Fixed rates of 1 × 10^−1^ and 1 × 10^−2^ initially converge rapidly but ultimately fail to train properly, as large learning rates cause overshooting and oscillate or even diverge near the loss minimum. While fixed rates of 1 × 10^−3^ and 1 × 10^−4^ enable stable training, they converge to suboptimal solutions, with 1 × 10^−3^ showing faster early-stage convergence but 1 × 10^−4^ reaching marginally better local optima later. Unlike fixed rates, adaptive learning rate strategies are more prevalent in deep learning. The Step Decay schedule in Figure 8 exhibits distinct plateaus and abrupt drops (particularly around epoch 40), whereas Exponential Decay and Cosine Decay display smoother trajectories. Notably, Cosine Decay achieves superior convergence by maintaining: (1) smoother, continuously differentiable rate transitions than Step Decay, and (2) more gradual decay than Exponential Decay, thereby prolonging effective optimization before premature stagnation. As quantified in Table 3, Cosine Decay performs better in most datasets, justifying its selection for subsequent experiments.

The fixed batch size is 4, and the learning rate adjustment strategy is Cosine Decay. Figure 8 shows the convergence of the model under different optimizers, and Table 4 shows the corresponding DR5.

Figure 8 demonstrates that SGD exhibits the slowest convergence due to its lack of adaptive learning rate adjustment. While AdaGrad converges relatively quickly, its training progress nearly stagnates after epoch 40 because it uses the accumulated sum of historical gradient squares as the denominator, causing the learning rate to decrease monotonically until it becomes infinitesimally small in later stages, halting parameter updates. Adam combines momentum with adaptive learning rates for faster convergence, whereas AdamW further improves upon Adam by decoupling weight decay to resolve conflicts between adaptive learning rates and L2 regularization, resulting in more stable training. As shown in Figure 8, AdamW slightly outperforms Adam in both convergence speed and stability. Table 4 confirms that AdamW achieves superior results on most datasets. Therefore, AdamW is selected as the optimizer for subsequent experiments.

After completing the ablation experiment of training hyperparameters, it is necessary to experimentally verify and select the hyperparameters of the model.

This paper compares two adjustable parameters in the experiment: patch size and the number of MSA modules, as well as the weight initialization scheme used in the weighted feature fusion method.

**Figure 9:**
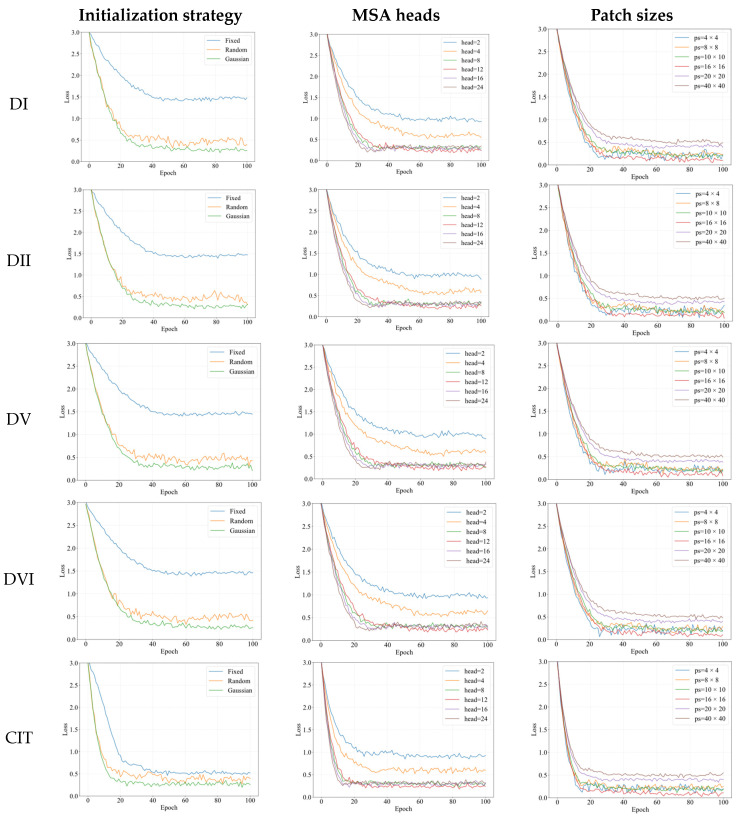
Demonstration of loss curves for ViMSA on partial datasets under different model hyperparameters.

The weighted feature fusion method’s performance is significantly affected by weight initialization, as it determines the learning direction of weight vectors. This paper systematically evaluates three distinct initialization approaches: fixed initialization where weights are set to a constant value of 1, random initialization with values uniformly distributed between 1 × 10^−5^ and 1, and Gaussian initialization where weights are randomly generated following a normal distribution centered on a 30 × 30-pixel region of the image with a standard deviation of 1. All experiments were performed using the default parameters of ViT and ViM models, maintaining a consistent patch size of 16 × 16 pixels and employing 8 MSA modules, with DR5 serving as the evaluation metric. The comprehensive comparison results are presented in Table 5. Figure 9 shows the convergence of the model under different weight initialization schemes.

As evidenced in Figure 9, the fixed initialization scheme yields the slowest convergence and even fails to converge normally on datasets like DI (with reflections), DII (poor illumination), and DV (contact lenses). This likely occurs because: (1) complex data distributions require differentiated feature learning, but fixed initialization forces identical neuron updates during backpropagation, reducing the network to single-neuron functionality; (2) while random initialization breaks symmetry and enables diversified feature learning, improperly configured random values may cause gradient instability and neuron saturation. In contrast, Gaussian initialization achieves the fastest convergence since: (a) its zero-centered symmetric weight distribution aligns with pupil image characteristics; (b) appropriate numerical ranges prevent activation saturation (e.g., ReLU outputs) during forward propagation, avoiding vanishing gradients; (c) uniform gradient distribution during backpropagation; and (d) random gaussian noise ensures differential initial weights for accelerated specialized learning. From Table 5, it can be seen that the Gaussian initialization scheme achieved better results on all datasets. Therefore, in the subsequent experiments, Gaussian initialization was chosen as the initialization scheme for the weights in the weighted feature fusion module.

Regarding patch size and the number of MSA modules, the original ViM model uses a 16 × 16 patch size, while the original ViT model employs 8 MSA heads. Set the patch size to 8 × 8, Figure 9 shows the loss curves of the model on different datasets under different MSA heads, and Table 6 shows the corresponding DR5.

Figure 9 demonstrates that when the number of MSA heads is too small (2 or 4 heads), convergence is slower and settles at suboptimal solutions due to insufficient capacity to capture the diversity and complexity in input data, limiting feature learning. Conversely, excessive heads (16 or 24) accelerate convergence but fail to escape poor local optima, as redundant heads may overfit to noise rather than meaningful patterns. Comparative analysis reveals that 12 heads strike an optimal balance, delivering moderate convergence speed and superior performance across most datasets. Thus, 12 MSA heads are selected for subsequent experiments.

Keeping the number of MSA heads fixed at 12, Figure 9 shows the loss curves of the model on different datasets for different patch sizes, and Table 7 shows the corresponding DR5.

Figure 9 demonstrates that when the patch size is too small (e.g., 4 × 4 or 8 × 8), the model converges faster but with relatively unstable training dynamics, as smaller patches preserve finer local details but are also more susceptible to local noise, resulting in larger gradient fluctuations. Conversely, excessively large patch sizes (e.g., 20 × 20 or 40 × 40) lead to more stable training but tend to converge to poorer local optima due to the loss of discriminative fine-grained features. The analysis reveals that a 16 × 16 patch size achieves an optimal balance, delivering faster convergence, moderate stability, and superior final performance. As evidenced in Table 7, the 16 × 16 configuration outperforms other settings across most datasets. Therefore, a patch size of 16 × 16 is selected for subsequent experiments.

### 4.5. Horizontal Experiment

This paper conducts a comparative analysis of both classical and state-of-the-art models in pupil detection. The evaluated models include: classical approaches—ExCuSe [25] (abbreviated as Ex), ELSe [26] (EL), PuReST [27] (PR), PupilNet v2.0 [35] (Pu, which contains three sub-models: direct approach SK8P8 (SK), direct coarse positioning and fine positioning implemented with FCKxPy (FC) and FSKxPy (FS)); and recent models from literature [15] (GC), [16] (KX), [17] (CS), [18] (ZHM), [19] (ZG), [20] (WL), [21] (JXM), [22] (ZB), [37] (GB), [38] (GY), along with ViT, ViM, ViM+MSA (VM), and ViT+FMSA (the proposed Fourier-accelerated MSA, denoted as VF). Table 8 and Table 9 summarize the RMSE values of classical and emerging models across all datasets, respectively, while Table 10 and Table 11 present the DR5 metrics for classical and emerging models on all datasets correspondingly.

The accuracy of models Ex, EL, Pu, and GB can be reused across some shared datasets. As shown in Table 8 and Table 9 (RMSE) versus Table 10 and Table 11 (DR5), classical pupil detection algorithms (Ex, EL, Pu) fail to deliver satisfactory results under varying illumination conditions. In contrast, the proposed ViMSA model maintains robust detection rates even under extreme conditions including poor lighting, reflections, contact lens, and mascara application, outperforming both classical image-processing-based methods and recently proposed detection algorithms in DR5 and RMSE metrics across most datasets. Notably, the SK sub-model of Pu achieves 99% accuracy and 1.7px RMSE on dataset DXVII, surpassing ViMSA’s performance—likely because this dataset contains only 268 images, whereas ViMSA (like other deep learning models) requires larger training sets to achieve optimal generalization, while the simpler SK architecture may perform better on smaller datasets. This suggests that expanding the pupil image volume could further improve ViMSA’s performance on such datasets. Table 9 and Table 11 demonstrates identical results between VM and ViMSA, as well as between VF and ViT, since their core computational workflows remain fundamentally unchanged, with FFT primarily serving to accelerate matrix multiplication.

**Figure 10:**
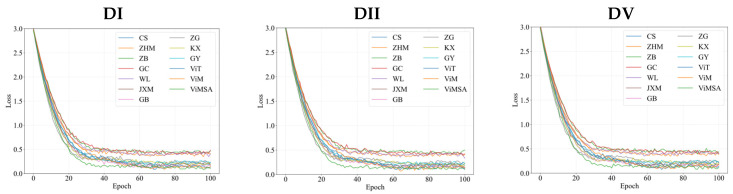
Comparison of loss curves of the latest pupil detection model on different datasets.

Figure 10 shows the loss function curves of the latest pupil detection model on different datasets.

From Figure 10, it can be seen that the convergence stability and efficiency of ViMSA are slightly better than other latest models.

Table 12 shows the GFLOPs and running time for each model.

As evidenced in Table 12, the proposed ViMSA model demonstrates superior computational efficiency compared to most state-of-the-art approaches, with a notably low complexity of 0.102 GFLOPs—surpassed only by the GB model and original ViM architecture. While GB achieves faster execution through its simplified dual-convolutional design (processing x/y coordinates separately), this decoupled approach fails to capture spatial correlations between coordinates, resulting in lower accuracy than ViMSA. The baseline ViM, though efficient, exhibits weaker global modeling capability versus ViT. ViMSA addresses this by integrating ViT’s MSA mechanism for enhanced global representation, achieving accuracy improvements with merely a 0.008 GFLOPs overhead. Experimental results confirm that replacing standard MSA with FFT-accelerated MSA in ViT (yielding VF) improves both speed and complexity. Ultimately, ViMSA meets real-time pupil detection requirements (>100 FPS) while maintaining robust performance across challenging conditions (poor illumination, occlusions etc.), as it combines: (1) ViM’s efficient SSM backbone, (2) MSA’s global attention, and (3) FFT optimization—collectively solving critical issues in extreme scenarios.

## 5. Discussion

The excellent detection performance of the proposed ViMSA model on multiple datasets indicates that the model can be independently used for all real-time eye tracking systems and gaze tracking systems that support head mounted pupil acquisition devices. Specifically, it can be applied to scenarios where the ambient light intensity changes rapidly, such as real-time gaze tracking between pilots, as well as pupil localization in strong light (frequent blinking) and pathological eyelid droop scenarios.

The conventional non-learning-based image processing algorithms (ELSe, ExCuSe, and PuReST) generally deliver satisfactory detection results, but exhibit severe performance degradation under specific illumination conditions as demonstrated in Table 9. Notably, the proposed ViMSA model achieves superior detection accuracy across all datasets, demonstrating significantly enhanced robustness to varying lighting conditions and partial occlusions compared to edge-detection and ellipse-fitting-based approaches like ELSe.

Compared with the CS, ZHM, WL, and ZG models, ZHM, CS integrates UNet with CSA, enhancing UNet’s capability to capture global features. However, the matrix operations involved in the CSA are computationally analogous to MSA, which inevitably increases the model’s computational complexity. WL employs a ResNeSt and ViT cascade as the backbone network, while ZHM adopts FPN combined with ViT in series as the backbone. Both architectures exhibit relatively high computational complexity, and their serialized network designs fail to fully leverage ViT’s global mutual information modeling capabilities. The ZG model uses the YOLOv8 object detection network as the backbone network, and increases the inter layer receptive field through the depth of the convolutional network to capture global information, however, the model has a high computational complexity. ViMSA has a simple structure and combines the global modeling capability of MSA with the efficiency of ViM, enabling efficient real-time pupil detection.

Compared with ZB, JXM, and GB models, JXM adopts a two-stage mode for detection. Although the model complexity is lower, it limits the further improvement of model detection accuracy because the two steps used for detection are not decoupled. ZB similarly adopts a two-stage paradigm, where the computational complexity remains non-negligible due to the employment of YOLOv5s for initial coarse localization in the first stage, while inheriting the inherent step-dependency limitations characteristic of two-stage architectures. GB decomposes pupil localization into x-direction localization and y-direction localization, ignoring the potential spatial correlation of pupil coordinates, which limits the improvement of model accuracy. ViMSA directly learns the relevant information of the pupil from the pupil image and simultaneously regresses the x and y coordinates without relying on other operations.

Compared with the GY model, the GY model uses ResNet as the backbone network and combines dilated convolution to adapt to images with different pupil sizes, but cannot escape the poor global modeling ability of CNN. ViM combines the global modeling capability of ViT with the efficiency of ViM, enabling real-time and efficient completion of pupil detection tasks.

Compared with the GC and KX model, The GC model incorporates the channel attention SE module to perform weighted calibration on the channel dimension of feature maps, while the KX model introduces a dual attention mechanism combining both spatial and channel attention, enabling adaptive learning of pupil features. However, its essence remains similar to the weighted feature fusion in ViMSA. In terms of model computational complexity, the weighted feature fusion method is significantly better than the attention mechanism.

Table 13 summarizes the aforementioned pupil detection approaches, listing their techniques, ability to capture global dependencies in pupil images, adaptive learning capacity, computational complexity, and inherent limitations.

In Table 13, the first column lists model names, the second column indicates the technical approaches of the models, and columns three to five present the three major advantages of a model in pupil detection tasks (√ denote the model possesses the corresponding advantage while blank spaces indicate its absence). The sixth column describes inherent limitations caused by certain models' training patterns or architectural designs.

One potential issue with the proposed model is that all datasets involved in the experiment have pupil image coordinate label pairs, which would cause the model to default to all input images containing pupils. For input images without pupils, a pseudo pupil coordinate will also be given during testing based on the internal information distribution of the image. A possible solution is to add a large number of images without pupils to the training data, such as completely closed eye images, environment images without human eyes, etc., so that the model learns patterns without pupil information and outputs empty coordinates that meet expectations.

In future research, this ViM-based pupil detection model can be used as an auxiliary technology for various real-time applications. In the medical industry, by tracking the movement trajectory of the subject’s pupils, it is possible to identify whether the subject has vestibular dysfunction. In the automotive industry, detecting driver distraction and identifying driver drowsiness based on eye tracking technology can improve driving safety. In addition, some educational platforms choose online education mode, allowing students to freely arrange their learning location and time, which can lead to lecturers being unable to track students’ attention in a timely manner. In this case, it is necessary to establish a VFOA tracking system to evaluate the effectiveness of the online learning environment.

## 6. Conclusions

This paper improves and extends ViM, proposing a new pupil center detection model ViMSA that can achieve high-precision and real-time pupil center localization under variable lighting conditions and partial occlusion.

In order to enable the model to correctly detect the center of the pupil in extreme situations, the proposed ViMSA model adopts two serial schemes. One is to introduce learnable weight parameters with the same number of patches, so that the model can learn the possible regions of pupils in the image in a targeted manner, similar to what attention mechanisms do, but with much lower computational complexity than the attention module. The second approach is to use ViM as the network backbone and integrate MSA, combined with ViT’s ability to capture global features in more detail, enabling the network to accurately detect pupil centers under different lighting and occlusion conditions. Based on this, FFT is used to further reduce the computational complexity of the model, forming the ViMSA model. Compared to other networks, the proposed ViMSA can achieve higher detection accuracy with lower computational complexity.

The proposed ViMSA model was evaluated on approximately 135,000 pupil images from 30 distinct datasets, comprising diverse samples captured under variable and non-uniform lighting conditions. These images have different reflections, covering eyelashes and eyebrows, using eye black or contact lenses.

Experimental results demonstrate that the proposed ViMSA achieves 99.6% DR5, with an RMSE of 1.67 pixels, while maintaining a processing speed exceeding 100 FPS, fulfilling real-time monitoring requirements for diverse applications.

Like other deep learning-based models, the performance of ViMSA depends on the quantity and diversity of the pupil image dataset used to train the network.

Based on these experimental results, the proposed ViMSA model is suitable for processing pupil images under various lighting and occlusion conditions in real-time systems due to its high accuracy and real-time performance. As expected, due to its good immunity to variable lighting conditions or the presence of some obstacles, its detection accuracy is significantly better than non-learning-based classical detection algorithms and existing learning-based network models.

## Figures and Tables

**Table 1:** Experimental hardware and operating environment parameters.

Hardware	Environmental Parameters
CPU	Intel(R) Core(TM) i7-14700HX CPU@2.10 GHz
GPU	8 GB NVIDIA GeForce GTX 4060
RAM	32 G
System	Windows 11
Language	Python3.11
DL Library	Pytorch2.6.0

**Table 2:** DR5 of ViMSA on different datasets under different batch sizes.

Dataset	Image Number	1	2	4	8	16	32	Description of the Dataset
DI	6554	75.04	75.97	80.58	81.89	79.39	81.78	Reflection
DII	505	73.84	78.05	82.79	80.11	80.7	79.8	Bad illumination
DIII	9799	83.99	76.93	84.53	79.81	79.54	81.33	Reflection
DIV	2655	73.49	79.27	84.08	82.8	79.69	79.74	Contact lenses
DV	2135	83.17	79.01	80.67	82.91	79.83	81.59	Shifted contact lenses
DVI	4400	83.01	76.14	83.49	80.45	75.27	80.18	Mascara
DVII	4890	73.58	83.48	81.84	79.3	77.57	80.48	Mascara, eyeshadow
DVIII	630	77.0	80.42	81.13	81.91	80.92	79.43	Eyelashes
DIX	2831	76.32	80.47	80.91	80.21	81.05	81.01	Additional black dot
DX	840	83.96	82.26	84.97	82.24	77.38	81.21	Bad illumination
DXI	655	72.94	81.3	82.32	80.53	80.5	81.25	Additional black dot
DXII	524	77.28	81.04	82.61	82.83	77.04	81.54	Bad illumination
DXIII	491	82.82	75.65	83.58	80.83	76.77	80.73	Bad illumination
DXIV	469	71.58	79.48	82.51	80.05	78.99	81.08	Bad illumination
DXV	363	74.15	79.57	82.19	81.15	76.3	80.73	Shifted contact lenses
DXVI	392	83.75	81.56	82.88	80.81	79.29	79.44	Mascara, eyeshadow
DXVII	268	80.01	76.76	80.83	82.42	75.37	80.96	Mascara
DXVIII	10,794	71.45	83.73	81.61	79.78	82.0	79.24	Reflection, Mascara
DXIX	13,474	73.19	75.13	84.67	80.72	77.65	79.87	Reflection
DXX	10,344	80.4	78.28	82.32	81.69	76.4	80.82	Reflection
DXXI	9133	72.18	76.29	84.59	80.85	77.3	80.74	Reflection, Bad illumination
DXXII	10,370	76.07	83.01	80.16	80.25	76.74	81.24	Reflection, Mascara
DXXIII	636	79.42	77.94	80.4	81.16	75.94	79.99	Bad illumination
DXXIV	961	76.6	75.21	80.87	80.66	77.68	79.13	Reflection
newDI	12,170	79.74	81.23	82.28	80.88	78.56	79.83	Reflection
newDII	7032	83.51	79.51	83.14	82.87	78.76	79.14	Reflection
newDIII	8780	71.1	75.23	81.95	80.53	78.1	80.57	Reflection, Bad illumination
newDIV	8691	72.5	83.3	84.41	79.55	76.01	81.22	Reflection
newDV	4544	74.9	78.14	83.35	80.96	76.21	80.24	Bad illumination
CIT	20,000	71.08	80.97	84.91	82.67	76.48	80.25	CASIA Iris Thousand

**Table 3:** DR5 of ViMSA on different datasets under different learning rates.

Dataset	1 × 10^−1^	1 × 10^−2^	1 × 10^−3^	1 × 10^−4^	1 × 10^−5^	Step Decay	Exponential Decay	Cosine Decay
DI	56.94	69.7	80.58	83.67	40.37	88.48	89.38	91.55
DII	28.73	70.45	82.79	86.12	29.84	87.0	89.31	93.17
DIII	39.36	50.25	84.53	85.17	40.57	87.87	91.12	90.55
DIV	28.88	43.24	84.08	86.64	57.81	89.63	88.75	90.47
DV	39.21	52.55	80.67	84.25	56.54	86.17	91.88	92.81
DVI	19.85	70.62	83.49	84.13	40.92	89.28	89.14	92.46
DVII	35.09	36.72	81.84	85.65	50.48	89.78	91.65	91.11
DVIII	33.06	63.91	81.13	82.16	51.29	88.41	90.99	93.18
DIX	20.51	41.23	80.91	83.92	47.62	87.73	91.84	93.96
DX	32.81	50.66	84.97	83.81	53.4	87.4	88.91	93.4
DXI	34.6	51.8	82.32	84.58	54.4	86.55	90.38	90.38
DXII	32.01	72.97	82.61	86.73	38.93	87.36	88.3	92.59
DXIII	33.45	56.21	83.58	82.07	27.91	88.97	88.95	93.29
DXIV	51.76	43.62	82.51	84.64	50.77	89.48	91.08	92.26
DXV	21.8	66.08	82.19	82.59	41.19	88.54	90.75	90.46
DXVI	44.04	37.16	82.88	83.0	54.8	89.29	90.33	92.47
DXVII	42.26	58.76	80.83	82.44	45.07	87.67	90.99	92.86
DXVIII	34.32	73.34	81.61	84.08	31.11	89.38	88.99	93.95
DXIX	57.57	53.72	84.67	84.81	47.51	87.79	91.58	91.61
DXX	26.15	62.7	82.32	86.16	53.16	86.35	88.4	90.75
DXXI	45.31	48.62	84.59	86.16	30.06	87.83	90.64	93.1
DXXII	25.97	61.04	80.16	84.66	29.9	88.37	90.32	90.57
DXXIII	35.27	65.73	80.4	84.77	33.67	89.99	88.34	92.38
DXXIV	55.57	60.79	80.87	85.98	46.2	89.69	89.35	93.06
newDI	49.29	60.29	82.28	85.37	58.18	86.6	88.43	92.3
newDII	41.42	51.11	83.14	84.26	42.62	89.61	88.02	93.26
newDIII	49.64	49.97	81.95	82.18	57.55	87.96	88.72	92.52
newDIV	45.27	42.9	84.41	83.57	47.18	86.79	89.4	93.34
newDV	56.58	53.72	83.35	86.36	55.49	89.76	88.63	91.88
CIT	56.74	59.55	84.91	85.92	23.42	88.21	90.95	90.47

**Table 4:** DR5 of ViMSA on different datasets under different optimizers.

Dataset	SGD	SGD with Momentum	AdaGrad	RMSProp	Adam	AdamW
DI	86.17	89.61	82.03	91.95	91.55	93.66
DII	87.78	89.87	85.75	89.35	93.17	92.6
DIII	86.14	88.85	78.28	88.11	90.55	92.26
DIV	87.1	87.57	79.62	89.24	90.47	91.69
DV	87.34	87.11	85.87	88.36	92.81	94.59
DVI	84.84	89.72	86.74	91.0	92.46	94.03
DVII	84.79	87.52	86.34	91.77	91.11	92.93
DVIII	85.33	87.52	85.55	91.88	93.18	93.79
DIX	85.31	89.62	83.59	91.61	93.96	91.62
DX	84.75	88.06	87.11	90.89	93.4	93.0
DXI	84.13	88.81	81.63	90.25	90.38	95.0
DXII	87.05	87.02	85.26	90.09	92.59	93.93
DXIII	87.87	87.45	84.3	91.79	93.29	94.54
DXIV	85.01	87.11	81.69	90.32	92.26	94.92
DXV	84.89	87.15	78.92	90.71	90.46	92.57
DXVI	85.68	88.23	85.73	91.57	92.47	93.45
DXVII	86.44	89.23	82.24	90.23	92.86	93.64
DXVIII	86.47	87.26	79.37	89.9	93.95	93.34
DXIX	84.53	88.55	80.91	89.31	91.61	93.28
DXX	84.48	87.5	82.19	91.86	90.75	93.0
DXXI	85.33	89.17	82.79	90.98	93.1	94.87
DXXII	87.41	87.33	79.65	88.76	90.57	92.57
DXXIII	86.39	87.48	80.61	90.1	92.38	93.4
DXXIV	87.65	87.55	82.43	91.76	93.06	92.11
newDI	86.61	89.9	81.88	88.57	92.3	94.72
newDII	87.2	88.5	86.6	90.72	93.26	94.13
newDIII	84.35	88.9	85.95	88.03	92.52	93.73
newDIV	84.17	88.72	83.17	91.51	93.34	91.18
newDV	84.02	87.1	86.0	88.12	91.88	92.86
CIT	85.76	89.92	80.81	88.3	90.47	93.66

**Table 5:** Weight initialization scheme and corresponding DR5.

Dataset	Fixed	Random	Gaussian
DI	54.32	84.96	93.66
DII	75.55	85.69	92.6
DIII	51.9	90.5	92.26
DIV	67.62	84.18	91.69
DV	59.76	90.05	94.59
DVI	54.41	84.1	94.03
DVII	61.8	88.2	92.93
DVIII	73.31	88.17	93.79
DIX	69.39	86.7	91.62
DX	58.08	86.95	93.0
DXI	74.35	88.33	95.0
DXII	57.48	87.94	93.93
DXIII	64.94	89.38	94.54
DXIV	72.92	89.63	94.92
DXV	52.34	90.85	92.57
DXVI	42.05	86.6	93.45
DXVII	54.29	90.26	93.64
DXVIII	45.55	90.55	93.34
DXIX	73.72	90.98	93.28
DXX	61.46	89.52	93.0
DXXI	50.58	87.62	94.87
DXXII	72.52	88.01	92.57
DXXIII	58.18	86.85	93.4
DXXIV	51.84	87.05	92.11
newDI	56.73	88.58	94.72
newDII	46.25	90.3	94.13
newDIII	46.85	85.42	93.73
newDIV	70.27	89.11	91.18
newDV	50.37	87.36	92.86
CIT	82.46	86.02	93.66

**Table 6:** Different MSA heads and corresponding DR5.

Dataset	2	4	8	12	16	24
DI	84.19	85.56	87.15	91.56	89.09	88.55
DII	85.34	86.79	87.18	90.67	85.36	82.92
DIII	85.67	86.21	87.14	91.03	89.58	88.92
DIV	85.32	84.43	86.55	86.46	86.71	87.77
DV	85.6	86.91	88.46	86.96	89.75	81.41
DVI	84.02	84.51	88.83	89.63	86.46	86.31
DVII	85.23	85.93	89.33	90.75	83.7	84.09
DVIII	84.71	84.1	88.95	90.95	85.15	82.83
DIX	85.99	85.41	88.15	90.82	89.96	89.86
DX	85.77	85.91	89.26	89.57	89.85	85.21
DXI	84.3	84.24	86.44	89.44	86.95	83.97
DXII	84.61	85.55	87.13	88.95	87.73	84.09
DXIII	85.12	85.25	90.7	86.12	88.42	89.73
DXIV	84.55	85.74	89.12	90.05	87.9	87.59
DXV	84.37	85.9	89.64	91.64	89.65	81.36
DXVI	85.08	86.21	87.44	86.75	85.31	85.9
DXVII	85.42	86.24	88.78	91.06	86.5	81.36
DXVIII	85.9	86.59	90.04	89.54	89.59	83.61
DXIX	85.4	84.89	89.82	91.52	90.91	83.35
DXX	85.05	84.76	88.54	88.74	89.56	84.56
DXXI	85.52	85.4	87.61	87.77	86.08	89.76
DXXII	85.88	86.69	89.33	90.23	88.51	84.59
DXXIII	84.01	84.08	89.07	86.59	83.46	87.16
DXXIV	85.49	85.87	90.73	91.48	90.31	85.59
newDI	85.69	84.54	89.97	91.69	84.69	88.02
newDII	85.52	84.78	87.95	92.7	88.25	85.87
newDIII	85.94	84.11	88.93	92.01	88.53	82.34
newDIV	85.64	86.16	86.16	91.43	88.65	86.57
newDV	84.67	85.96	90.87	86.26	89.04	84.27
CIT	85.38	85.9	90.69	92.93	90.37	87.12

**Table 7:** Different patch sizes and corresponding DR5.

Dataset	4 × 4	8 × 8	10 × 10	16 × 16	20 × 20	40 × 40
DI	83.32	91.56	93.18	96.69	89.58	86.14
DII	85.42	90.67	90.16	95.96	89.42	87.48
DIII	83.06	91.03	96.91	93.88	87.78	87.96
DIV	85.85	86.46	91.63	98.07	88.48	86.62
DV	84.47	86.96	94.77	98.68	88.56	86.87
DVI	83.17	89.63	90.83	98.5	89.15	87.33
DVII	85.15	90.75	95.63	94.9	87.1	85.69
DVIII	88.9	90.95	96.76	98.46	89.73	85.6
DIX	86.2	90.82	96.73	98.03	89.39	85.48
DX	89.09	89.57	90.12	96.38	87.04	86.05
DXI	88.21	89.44	94.77	93.59	88.84	86.63
DXII	91.37	88.95	95.58	97.33	88.69	86.46
DXIII	85.29	86.12	90.83	95.66	87.43	87.17
DXIV	84.89	90.05	94.45	97.48	88.06	86.82
DXV	83.46	91.64	91.46	98.42	88.25	85.52
DXVI	86.48	86.75	95.65	96.86	89.92	86.52
DXVII	90.96	91.06	90.81	98.14	87.98	86.21
DXVIII	85.24	89.54	91.37	95.98	89.88	86.22
DXIX	90.15	91.52	94.3	97.57	87.21	85.94
DXX	91.58	88.74	90.47	96.7	88.01	87.25
DXXI	83.96	87.77	96.67	97.86	88.92	86.02
DXXII	84.39	90.23	96.46	93.4	88.12	85.13
DXXIII	89.93	86.59	94.52	95.63	87.02	85.21
DXXIV	85.23	91.48	92.28	93.37	87.02	86.4
newDI	89.59	91.69	91.19	98.94	87.4	85.95
newDII	83.44	92.7	95.09	95.94	87.96	86.18
newDIII	83.25	92.01	90.12	93.06	88.35	86.65
newDIV	89.76	91.43	94.09	95.7	89.48	85.72
newDV	87.86	86.26	94.58	96.59	87.36	86.92
CIT	92.02	92.93	97.21	99.6	90.36	89.2

**Table 8:** RMSE of various classic models on different datasets.

DB	Ex	EL	PR	Pu			Ours
				SK	FC	FS	
DI	6.8	4	4.7	5.8	5.6	4.9	2.14
DII	13	8	7.3	5.3	5.4	5.4	2.28
DIII	13	8	7.7	8.6	9	7.9	2.67
DIV	5.3	5	3.8	3.4	3.4	3	1.88
DV	6	4	3.6	3.2	3.6	3	1.77
DVI	9	6	6.2	6.6	5.6	5.4	1.8
DVII	11	9	8.3	6.6	5.3	6.6	2.47
DVIII	9.9	7	6.6	4.5	4.7	5.1	1.81
DIX	6	4	4.1	4.1	4.1	4.1	1.89
DX	5.4	5	5.3	5.3	5.6	5.1	2.2
DXI	9.4	6	4.5	4.3	6.4	3.2	2.72
DXII	5.3	5	4.9	4	4.3	4.3	2.02
DXIII	7.3	6	5.1	5.4	5.1	4.7	2.33
DXIV	7.5	4	4	3.2	2.6	2.5	1.99
DXV	9.7	9	6.8	5.1	6.9	5.1	1.82
DXVI	14	9	7.3	5.3	6.8	5.3	2.11
DXVII	5.4	3	2.8	1.7	4.0	2.1	1.87
DXVIII	15	10	9.2	9.9	12	8.6	2.27
DXIX	16	14	13	14	16	13	1.98
DXX	9.4	5	6.4	5.4	6.6	5.4	2.14
DXXI	10	11	8	5.1	7.7	4.7	1.92
DXXII	15	10	10	11	10	9.4	2.76
DXXIII	2.8	3	3	4.1	4.0	3.4	2.34
DXXIV	12	10	9.9	11	9.9	9.9	2.76
newDI	16	8	8.3	7.3	9.7	7.3	1.72
newDII	17	15	14	12	14	12	2.28
newDIII	14	13	13	12	12	11	2.82
newDIV	11	10	7.7	4.7	5.8	5	2.33
newDV	9.2	6	5.4	5.6	6	5	2.16
CIT	4.7	4	3.6	3	2.6	2.6	1.67

**Table 9:** RMSE of emerging models on different datasets.

DB	CS	ZHM	ZB	GC	WL	JXM	GB	ZG	KX	GY	ViT	ViM	VM	VF	Ours
DI	2.96	4.22	4.9	4.09	4.6	2.4	2.4	2.5	3.0	3.3	2.8	3.1	2.14	2.8	2.14
DII	2.86	3.32	4.57	3.69	4.8	3.3	3	2.6	3.1	3.6	2.9	2.7	2.28	2.9	2.28
DIII	2.26	3.77	3.15	3.86	3.2	3.3	1.9	3.1	2.3	3.3	3.1	2.6	2.67	3.1	2.67
DIV	2.5	3.06	5.14	4.94	3.7	2.6	2	2.4	3.4	3.5	3.2	2.6	1.88	3.2	1.88
DV	2.66	3.74	3.89	3.56	3.1	2.2	2	2	2.4	2.9	2.6	2.8	1.77	2.6	1.77
DVI	2.44	4.26	3.93	3.06	3.1	2.7	2.1	2.7	2.4	2.6	2.6	2.8	1.8	2.6	1.8
DVII	2.13	4.33	2.79	2.91	4.5	2.6	2.8	2.7	2.4	3.8	2.4	2.7	2.47	2.4	2.47
DVIII	2.34	4.19	3.57	3.59	3.5	3.1	2.6	3.1	2.8	3	3.5	3	1.81	3.5	1.81
DIX	2.7	4.4	3.71	3.4	3	2.6	2.1	2.3	2.9	2.8	3.4	2.8	1.89	3.4	1.89
DX	2.63	3.9	3.84	4.49	4.1	3.3	2.3	3	2.5	4.5	2.7	2.5	2.2	2.7	2.2
DXI	3.0	2.67	5.21	4.2	4.2	2.5	1.8	1.8	3	3.1	3.2	2.8	2.72	3.2	2.72
DXII	2.67	4.52	4.94	4.98	4.6	2.3	2.7	2.4	2.6	3.1	3.2	3.1	2.02	3.2	2.02
DXIII	2.98	2.99	3.13	4.92	4.3	2.9	3.1	3.1	2.6	2.9	3.3	3.3	2.33	3.3	2.33
DXIV	2.48	3.9	3.36	5.06	3.8	3.1	2.5	2.9	3	2.5	3.5	2.7	1.99	3.5	1.99
DXV	2.39	3.73	4.85	3.98	3	2.5	2.2	1.9	3.1	3.2	3.3	3.4	1.82	3.3	1.82
DXVI	3.09	4.37	2.81	3.56	4.5	2.2	3.4	2.5	3.1	3.8	3.2	3	2.11	3.2	2.11
DXVII	2.72	3.87	4.12	3.86	2.9	2.4	2.1	3.2	3	4.0	3.4	2.7	1.87	3.4	1.87
DXVIII	3.38	2.8	4.04	4.22	4.1	3	3.2	2.9	3	4.5	3.1	2.8	2.27	3.1	2.27
DXIX	3.14	4.34	3.76	4.73	3.4	3.3	2.4	2.2	2.4	3.8	3	2.8	1.98	3	1.98
DXX	2.17	4.59	3.61	5.03	4.4	2.9	2.5	2.2	3.1	2.8	2.8	3.2	2.14	2.8	2.14
DXXI	2.78	2.72	3.21	3.87	3.2	2.8	2.1	2.1	2.5	4.1	3	3.4	1.92	3	1.92
DXXII	2.73	3.61	4.15	4.3	4.1	2.1	3.2	2.2	3	3.7	2.4	2.7	2.76	2.4	2.76
DXXIII	2.84	3.15	3.21	3.07	3.1	3.1	2.6	2.6	3.5	3.6	3	2.5	2.34	3	2.34
DXXIV	2.08	3.94	4.04	3.62	3.4	3.2	2.4	2.5	3.6	3.2	2.4	3	2.76	2.4	2.76
newDI	2.56	4.67	3.65	3.81	3.4	3.1	2.4	3.2	3.5	4.1	2.4	2.5	1.72	2.4	1.72
newDII	2.94	3.98	4.17	4.69	4	3	2.5	2.9	2.9	3.8	2.3	2.7	2.28	2.3	2.28
newDIII	3.07	3.64	5.08	4.99	3.3	3	2.1	2.0	3.3	4	3.6	3.2	2.82	3.6	2.82
newDIV	2.71	4.15	2.82	3.2	4.6	2.4	2.3	3.1	2.7	3.5	2.7	2.6	2.33	2.7	2.33
newDV	2.58	3.71	4.67	4.4	4.1	2.8	2.5	3	3.5	3	3.4	2.5	2.16	3.4	2.16
CIT	1.89	2.51	2.82	2.99	2.7	1.8	2	1.9	2.2	3.9	3.2	2.8	1.67	3.2	1.67

**Table 10:** DR5 of various classic models on different datasets.

DB	Ex	EL	PR	Pu			Ours
				SK	FC	FS	
DI	72	86	83	77	78	82	96.69
DII	40	65	69	80	79	79	95.96
DIII	38	64	67	62	60	66	93.88
DIV	80	83	88	90	90	92	98.07
DV	76	85	89	91	89	92	98.68
DVI	60	78	75	73	78	79	98.5
DVII	49	60	64	73	80	73	94.9
DVIII	55	68	73	84	83	81	98.46
DIX	76	87	86	86	86	86	98.03
DX	79	79	80	80	78	81	96.38
DXI	58	75	84	85	74	91	93.59
DXII	80	79	82	87	85	85	97.33
DXIII	69	74	81	79	81	83	95.66
DXIV	68	84	87	91	94	95	97.48
DXV	56	57	72	81	71	81	98.42
DXVI	35	60	69	80	72	80	96.86
DXVII	79	90	93	99	87	97	98.14
DXVIII	24	57	59	55	44	62	95.98
DXIX	23	33	36	34	20	37	97.57
DXX	58	78	74	79	73	79	96.7
DXXI	52	47	65	81	67	83	97.86
DXXII	26	53	54	50	52	58	93.4
DXXIII	93	94	92	86	87	90	95.63
DXXIV	46	53	55	46	55	55	93.37
newDI	22	62	64	69	56	69	98.94
newDII	16	26	33	44	35	45	95.94
newDIII	34	39	38	45	44	49	93.06
newDIV	48	54	67	83	77	82	95.7
newDV	59	75	79	78	76	81	96.59
CIT	83	85	89	92	94	94	99.6

**Table 11:** DR5 of emerging models on different datasets.

DB	CS	ZHM	ZB	GC	WL	JXM	GB	ZG	KX	GY	ViT	ViM	VM	VF	Ours
DI	92.31	85.55	81.92	86.25	83.79	95.33	95.51	94.51	92.21	90.75	93.09	91.57	96.69	93.09	96.69
DII	92.83	90.36	83.72	88.39	82.61	90.7	92.27	94.44	91.46	88.73	92.42	93.64	95.96	92.42	95.96
DIII	96.06	87.96	91.29	87.47	91.03	90.4	97.97	91.38	95.9	90.29	91.62	94.33	93.88	91.62	93.88
DIV	94.74	91.76	80.65	81.71	88.25	94.35	97.36	95.21	89.75	89.27	90.8	94.0	98.07	90.8	98.07
DV	93.91	88.16	87.34	89.12	91.75	96.57	97.56	97.4	95.23	92.53	94.16	92.99	98.68	94.16	98.68
DVI	95.06	85.34	87.13	91.78	91.48	93.92	96.88	93.46	95.37	94.39	94.43	93.12	98.5	94.43	98.5
DVII	96.74	84.97	93.23	92.56	84.09	94.37	93.29	93.87	95.29	87.74	95.36	93.6	94.9	95.36	94.9
DVIII	95.6	85.72	89.06	88.93	89.26	91.42	94.44	91.82	93.06	92.33	89.2	91.92	98.46	89.2	98.46
DIX	93.69	84.63	88.31	89.94	92.0	94.15	97.03	95.84	92.48	93.42	89.9	92.95	98.03	89.9	98.03
DX	94.08	87.3	87.62	84.1	86.06	90.4	95.87	91.91	94.5	84.2	93.84	94.87	96.38	93.84	96.38
DXI	92.09	93.87	80.27	85.65	85.5	94.73	98.47	98.46	92.12	91.54	91.25	92.97	93.59	91.25	93.59
DXII	93.86	83.97	81.72	81.5	83.28	95.78	93.85	95.22	94.17	91.41	90.87	91.37	97.33	90.87	97.33
DXIII	92.21	92.15	91.38	81.81	85.11	92.55	91.64	91.49	93.99	92.48	90.24	90.34	95.66	90.24	95.66
DXIV	94.85	87.28	90.14	81.07	88.03	91.69	94.66	92.53	92.2	94.79	89.29	93.44	97.48	89.29	97.48
DXV	95.35	88.17	82.18	86.87	92.16	94.65	96.41	97.88	91.64	91.09	90.28	90.13	98.42	90.28	98.42
DXVI	91.59	84.74	93.1	89.1	84.02	96.43	90.05	94.69	91.7	87.93	91.19	92.06	96.86	91.19	96.86
DXVII	93.58	87.43	86.09	87.49	92.57	95.15	96.64	90.79	92.09	86.68	89.85	93.74	98.14	89.85	98.14
DXVIII	90.06	93.15	86.52	85.58	86.28	91.97	91.25	92.39	92.27	84.32	91.7	92.98	95.98	91.7	95.98
DXIX	91.33	84.94	88.03	82.84	89.73	90.32	95.21	96.27	95.28	87.8	92.19	93.2	97.57	92.19	97.57
DXX	96.53	83.59	88.84	81.21	84.85	92.43	94.93	96.26	91.69	93.29	93.16	91.19	96.7	93.16	96.7
DXXI	93.29	93.61	90.98	87.42	91.17	93.22	96.97	97.14	94.88	86.08	92.12	90.16	97.86	92.12	97.86
DXXII	93.54	88.85	85.93	85.13	86.16	97.16	90.89	96.12	91.9	88.45	95.17	93.6	93.4	95.17	93.4
DXXIII	92.97	91.28	90.98	91.7	91.53	91.55	94.11	94.15	89.24	88.77	92.22	94.85	95.63	92.22	95.63
DXXIV	97.01	87.08	86.51	88.77	90.06	90.92	95.32	94.66	88.95	91.03	95.31	92.15	93.37	95.31	93.37
newDI	94.46	83.16	88.61	87.75	89.81	91.5	95.27	91.02	89.21	85.99	95.39	94.79	98.94	95.39	98.94
newDII	92.41	86.84	85.84	83.06	86.62	91.95	94.65	92.65	92.75	87.99	95.94	93.84	95.94	95.94	95.94
newDIII	91.7	88.67	80.96	81.44	90.52	91.86	96.72	97.55	90.4	86.54	89.1	91.25	93.06	89.1	93.06
newDIV	93.62	85.92	93.06	91.0	83.62	95.3	96.02	91.81	93.75	89.62	93.8	94.32	95.7	93.8	95.7
newDV	94.31	88.27	83.18	84.63	86.38	92.95	94.58	91.83	89.65	91.98	90.18	94.69	96.59	90.18	96.59
CIT	98.04	94.71	93.04	92.15	93.45	98.44	97.68	98.24	96.5	87.51	91.22	93.16	99.6	91.22	99.6

**Table 12:** The GFLOPs and running time for each model.

Model	GFLOPs(G)	Running Time (ms)
ExCuSe	-	6
ELSe	-	7
PuReST	-	4
Pu (SK)	-	7
Pu (FC)	-	1200
Pu (FS)	-	850
CS	14.61	43
ZHM	15.35	55
WL	16.81	57
JXM	0.143	10
GB	0.093	8
GC	4.4	17.6
ZG	11.2	31
ZB	6.4	24
KX	4.2	18.37
GY	4.1	17
ViT	17.6	52
ViM	0.0941	8
VM	0.32	49
VF	15.39	21
ViMSA	0.102	9

**Table 13:** Summary of various models.

	Technologies	Global Dependencies	Adaptive Learning	Low Complexity	Limitations
CS	Improve self attention mechanism	√			
ZHM	Integrating FPN and ViT	√			
WL	Integrating ResNeSt and ViT	√			
ZG	Introduce deformable convolution, deepen the model depth	√	√		
ZB	YOLOv5s coarse localization, Canny edge detection and fine localization				Step dependency
JXM	CNN two-step voting mechanism			√	Step dependency
GB	Decoupling x, y coordinates			√	Ignore the intrinsic connection between x and y
GC	Introducing SE module		√		
KX	Introducing a dual attention mechanism		√		
GY	Introducing dilated convolution		√		
Ours	Improve ViM, introducing weighted feature fusion, accelerate MSA	√	√	√	

## Data Availability

The dataset used in the article can be found on the website https://es-cloud.cs.uni-tuebingen.de/d/8e2ab8c3fdd444e1a135/?p=/datasets-head-mounted&mode=list, listed in reference [48].
